# Frequency-Specific Synchronization in the Bilateral Subthalamic Nuclei Depending on Voluntary Muscle Contraction and Relaxation in Patients with Parkinson’s Disease

**DOI:** 10.3389/fnhum.2016.00131

**Published:** 2016-03-30

**Authors:** Kenji Kato, Fusako Yokochi, Hirokazu Iwamuro, Takashi Kawasaki, Kohichi Hamada, Ayako Isoo, Katsuo Kimura, Ryoichi Okiyama, Makoto Taniguchi, Junichi Ushiba

**Affiliations:** ^1^Department of Neurology, Tokyo Metropolitan Neurological HospitalTokyo, Japan; ^2^Department of Biosciences and Informatics, Faculty of Science and Technology, Keio UniversityKanagawa, Japan; ^3^Department of Neurosurgery, Tokyo Metropolitan Neurological HospitalTokyo, Japan

**Keywords:** subthalamic nucleus, Parkinson’s disease, neuronal oscillations, coherence, cross-frequency coupling, deep brain stimulation

## Abstract

The volitional control of muscle contraction and relaxation is a fundamental component of human motor activity, but how the processing of the subcortical networks, including the subthalamic nucleus (STN), is involved in voluntary muscle contraction (VMC) and voluntary muscle relaxation (VMR) remains unclear. In this study, local field potentials (LFPs) of bilateral STNs were recorded in patients with Parkinson’s disease (PD) while performing externally paced VMC and VMR tasks of the unilateral wrist extensor muscle. The VMC- or VMR-related oscillatory activities and their functional couplings were investigated over the theta (4–7 Hz), alpha (8–13 Hz), beta (14–35 Hz), and gamma (40–100 Hz) frequency bands. Alpha and beta desynchronizations were observed in bilateral STNs at the onset of both VMC and VMR tasks. On the other hand, theta and gamma synchronizations were prominent in bilateral STNs specifically at the onset of the VMC task. In particular, just after VMC, theta functional coupling between the bilateral STNs increased, and the theta phase became coupled to the gamma amplitude within the contralateral STN in a phase-amplitude cross-frequency coupled manner. On the other hand, the prominent beta-gamma cross-frequency couplings observed in the bilateral STNs at rest were reduced by the VMC and VMR tasks. These results suggest that STNs are bilaterally involved in the different performances of muscle contraction and relaxation through the theta-gamma and beta-gamma networks between bilateral STNs in patients with PD.

## Introduction

In the execution of volitional movement in the human motor repertoire, not only voluntary muscle contraction (VMC), but also voluntary muscle relaxation (VMR) is a fundamental component. Previous electrophysiological studies with electroencephalograms (EEGs) in healthy participants reported that muscle relaxation is preceded by a cortical preparatory activity at the primary motor cortex and supplementary motor areas (Terada et al., 1995, 1999; Rothwell et al., 1998; Alegre et al., 2003). Imaging studies with functional magnetic resonance imaging have also demonstrated an increase in the blood-oxygen level-dependent signal, at least in the primary motor cortex and supplementary motor areas, during VMR, probably through the contribution of corticospinal tracts targeting spinal inhibitory interneurons or inhibitory cortical neurons (Terada et al., 1995; Toma et al., 1999; Pope et al., 2007). Despite this evidence for cortical involvement in VMR, the details of the subcortical neural mechanisms of VMR remain largely unknown.

The subthalamic nucleus (STN), which is one of the key subcortical motor centers mediating the cortico-basal ganglia-thalamo-cortical loop, is well known to play an important role in voluntary movements such as VMC and VMR. Previous electrophysiological studies investigating STN neurons in the monkey during arm movements have found that some neurons show an increased discharge rate, and other neurons show a decreased discharge rate with increased movement amplitude (Georgopoulos et al., 1983). Although large variabilities in positive or negative correlations between the discharge rate and movement amplitude were observed, consistent results from monkey neural recording and human imaging studies have demonstrated that subthalamic activities are scaled with the dynamic parameters of grip force output such as amplitude and rates (DeLong et al., 1984; Spraker et al., 2007; Prodoehl et al., 2009). On the other hand, the STN has also been implicated in suppressing an initiated go response in a stop signal response task (Aron and Poldrack, 2006). This is probably because subthalamic activation leads to a broad inhibition of thalamocortical projections, resulting in a global movement inhibition for stopping impulsive responses (Aron, 2011). Considering previous findings, the STN may be involved in both movement excitation and inhibition. However, the characteristics of human STNs in the fundamental domain of movements such as VMC and VMR are still unknown.

Recently, the characteristics of human STNs has been intensively demonstrated by investigating the movement-related local field potentials (LFPs) recorded from the STN through electrodes implanted for deep brain stimulation (DBS) to treat Parkinson’s disease (PD; Kühn et al., 2004; Loukas and Brown, 2004; Androulidakis et al., 2007; Kempf et al., 2007; Ray et al., 2012). Utilizing this opportunity, previous studies have shown that the magnitude of alpha (8–13 Hz) and beta (14–35 Hz) bands in the STN decreases in response to VMC in terms of event-related desynchronization (ERD), and following that, an increase occurs in these frequencies in terms of event-related synchronization (ERS; Kühn et al., 2004; Ray et al., 2012). On the other hand, gamma ERS (40–100 Hz) is also simultaneously observed after movement initiation (Cassidy et al., 2002; Alonso-Frech et al., 2006; Devos and Defebvre, 2006; Androulidakis et al., 2007). These frequency-specific movement-related ERD and ERS occur in the two STNs across hemispheres (Alegre et al., 2005), suggesting that bilateral STNs are involved with the initiation of a unilateral motor program. In addition, the alpha functional coupling between bilateral STNs increases prior to the onset of unilateral movement (Darvas and Hebb, 2014). This finding suggests that frequency-specific bilateral networks between the two STNs are activated for a unilateral motor program of VMC. As for the subcortical role in VMR, one previous study reported that the alpha/beta ERD and subsequent ERS recorded from the contralateral STN occur in both VMC and VMR tasks in patients with PD (Hsu et al., 2012; Tan et al., 2013). However, how functional coupling is bilaterally activated through the different VMC and VMR tasks is largely unclear.

Moreover, recent studies have demonstrated that functional couplings between different frequency bands, in terms of phase-amplitude cross-frequency coupling (PAC), may play a physiologically fundamental role in regulation of motor behavior (de Hemptinne et al., 2013; von Nicolai et al., 2014). In the “OFF” medication state in patients with PD, gamma oscillation is entrained by the phase of excessive beta oscillation in the STN and sensorimotor cortex (López-Azcárate et al., 2010; Yang et al., 2014; de Hemptinne et al., 2013, 2015). This beta-gamma PAC is reduced by medication (López-Azcárate et al., 2010), DBS treatment (de Hemptinne et al., 2013, 2015), and voluntary movement (de Hemptinne et al., 2013, 2015), suggesting that it may reflect the neural constrained states of an inflexible pattern of corticosubthalamic activities, leading to parkinsonian motor symptoms such as bradykinesia and rigidity. On the other hand, other researchers have demonstrated that in the striatum of healthy rats, increased gamma activities during motor behavior are entrained not by the beta phase but the theta phase, suggesting that the theta-gamma PAC may be directly related to the functional domain of motor behavior in the cortico-basal ganglia system (von Nicolai et al., 2014).

From these previous findings, frequency-specific subthalamic activities and their functional couplings, in terms of coherence or PAC, are differently involved in VMC and VMR, although the details of the distinct characteristics of each frequency component (i.e., theta, alpha, beta, and gamma bands) are largely unknown. Our motivation for this study was to clarify the distinct features of the subthalamic oscillations and their functional couplings over the theta, alpha, beta, and gamma ranges during the different motor controls of VMC and VMR in patients with PD. In this study, VMC- and VMR-related synchronization and desynchronization in the bilateral STNs were investigated when patients were performing externally paced VMC and VMR tasks of unilateral wrist extensor muscles. Coherence was analyzed to evaluate the movement-related functional coupling between the bilateral STNs in response to VMC and VMR tasks. Moreover, the PAC was analyzed to investigate the cross-frequency interaction in the bilateral STNs depending on the different motor processes of the VMC and VMR tasks.

## Materials and Methods

### Participants and Surgical Procedure

The participants were seven right-handed PD patients (4 males and 3 females; age, 57.0 ± 8.6 years; disease duration, 15.2 ± 5.3 years) undergoing surgery for implantation of DBS electrodes into the STN. These patients corresponded to Patients 4, 5, 6, 7, 8, 10, and 11 in the previous work by Kato et al. (2015). All patients received bilateral electrodes. The present study was approved by the ethics committees of the Tokyo Metropolitan Neurological Hospital (Tokyo, Japan) and the Faculty of Science and Technology, Keio University, Kanagawa, Japan. All patients provided their written informed consent before the recording. Each patient was diagnosed as having PD by the UK PD Society Brain Bank clinical diagnostic criteria (Daniel and Lees, 1993). Stereotactic surgery was performed according to the following procedures reported previously (Kato et al., 2015). Patients were withdrawn from their anti-Parkinsonian medication at least 12 h prior to the recordings, and the recordings were performed with the patients in the “OFF” medication state. The details of medication and Unified PD rating scale (UPDRS) scores in each patient are summarized in Table 1.

**Table 1:** **Clinical characteristics of Parkinson’s disease (PD) patients**.

				UPDRS III Total score	
Patient number	Sex	Age (years)	Disease duration (years)	ON	OFF	LED (mg)
1	F	45	8	23	36	452
2	M	47	9	29	38	266
3	M	65	16	16	34	702
4	M	58	14	20	37	695
5	M	48	22	14	24	1295
6	F	61	11	1	28	1196
7	F	65	20	30	41	716

*UPDRS: Unified Parkinson’s disease rating scale, On: on phase of wearing-off phenomenon, Off: off phase of wearing-off phenomenon, LED: Levodopa equivalent dose. LED 100 mg = 100 mg standard levodopa = 133 mg of controlled-release levodopa = 10 mg bromocriptine = 1 mg pergolide = 1 mg premipexole = 5 mg ropinirole*.

### Local Field Potentials and Electromyogram (EMG) Recordings

LFPs in bilateral STNs were recorded from the four contacts of each DBS electrode (Model 3389; Medtronic Neurological Division, Minneapolis, MN, USA) referenced to linked ears 2 days after the operation. The surface electromyogram (EMG) was simultaneously recorded bilaterally using pairs of Ag/AgCl disk electrodes (10 mm diameter) attached to the muscle bellies (20 mm interelectrode distance) of the following muscles: sternocleidomastoid, biceps brachii, triceps brachii, flexor carpi radialis, and extensor carpi radialis. Then, LFPs and EMG data were band-pass filtered (LFP data, 0.5–400 Hz; EMG data, 20–400 Hz) and digitized (sampling frequency, 1000 Hz) using a biosignal recorder (Neurofax EEG 1200; Nihon Kohden Corporation, Tokyo, Japan). Data were analyzed using Matlab (Mathworks, Natick, MA, USA). STN LFPs were calculated for DBS contact pairs 0–1, 1–2, and 2–3 by subtracting the signal of the contact pair from the signal of the adjacent contact pair.

### Movement Task

Patients were awake and instructed to lie in the supine position with their eyes closed. To confirm that patients made no involuntary movements during the task, the EMG was monitored in all patients throughout the experiments. Taking into account the feasibility of the movement task, each patient performed the movement task using the less-affected side (4 patients on the right side; 3 patients on the left side). They were asked to perform tonic extension and relaxation of the wrist. After an auditory cue, they dorsiflexed the wrist as quickly as possible and sustained the posture by contracting the wrist extensor muscles for 10 s. The duration of the auditory cue for the initiation of VMC was 1 s. During this phase, the patients were instructed not to contract muscles in other limbs. After the VMC phase for 10 s, a sound in another pitch was generated, and the patients were then instructed to place the palm back into the original flat position by relaxing their wrist muscles as soon as possible. The duration of the auditory cue for the initiation of VMR was 1 s. VMC and VMR sessions were repeated over 30 trials. The onset of muscle contraction and relaxation was defined as the time to exceed five standard deviations (SD) of the baseline muscle activities, averaged from 4 s to 2 s before the VMC onset. In six out of seven patients, the timing of the cue signal was simultaneously recorded, and the response time of muscle contraction and relaxation was calculated for each patient.

### Power Spectral Analysis

To evaluate the frequency-specific power changes in bilateral STNs, the discrete Fourier transform, calculated in accordance with the method of Halliday et al. (1995), was used for power spectral analysis. The LFP data recorded from the adjacent contacts of the electrodes (i.e., contacts 0–1, 1–2, and 2–3) for each trial were segmented into epochs of 1024 data points of the same duration of 1.024 s, yielding a frequency-resolution of 0.987 Hz. Each data segment was Hanning-windowed to reduce spectral leakage. The VMC- or VMR-related oscillatory changes in the bilateral STNs were investigated by computing the time-frequency map of the power spectrum over a sliding short-time (1.024 s) window moved in 0.05 s steps. As for the time periods for the analysis of power changes in the VMC and VMR tasks, 8 s durations ranging from 4 s before to 4 s after the VMC or VMR task were extracted in each task. Movement-related power was then averaged across contacts and trials, and the percentage values were calculated in relation to the baseline period, which was defined as 100%, ranging from 4 s to 2 s before the VMC.

The frequency range for the latter quantitative analyses was set at 2–100 Hz, including the theta (4–7 Hz), alpha (8–13 Hz), beta (14–35 Hz), and gamma (40–100 Hz) bands. The periods used in the statistical procedure were segmented as follows: from 0 s to 1 s after the VMC and VMR as the duration of the movement, and from 2 s to 4 s after VMC as the duration of holding VMC. Movement-related power changes in the respective frequency bands were assessed using factorial analysis of variance (ANOVA) with Bonferroni adjustment for multiple comparisons. The four factors were patient, period (i.e., 0–1 s after VMC, 2–4 s after VMC, and 0–1 s after VMR), side (i.e., ipsilateral and contralateral sides), and frequency (i.e., theta, alpha, beta, and gamma bands).

### PAC

To investigate the cross-frequency interaction during the VMC and VMR tasks, PAC between phase values of the low frequency including the theta, alpha, and beta ranges and the amplitude value of high frequency of the gamma range components was evaluated based on the methods developed by Canolty et al. (2006). The amplitude of phase-amplitude coupling was determined by computing a phase-modulation-index (MI) based on mutual information as described in Hurtado et al. (2004) and Tort et al. (2010). The periods used in the statistical procedure were segmented as follows: from −2 s to −1 s of the VMC onset as the duration of the resting state, from 0 s to 1 s after the VMC and VMR as the duration of the movement, and from 2 s to 4 s after VMC as the duration of holding VMC. Then, the phase-MI was averaged across contacts and patients. Movement-related changes in the phase-MI were assessed using factorial ANOVA with Bonferroni adjustment for multiple comparisons. The four factors were patient, period, side, and frequency.

### Coherence

To investigate the functional coupling between the bilateral STNs in the VMC and VMR tasks, coherence (Rosenberg et al., 1989; Halliday et al., 1995; Ushiyama and Ushiba, 2013; Kato et al., 2015) was evaluated between the following couplings: left STN contacts 3–2 vs. right STN contacts 3–2, left STN contacts 2–1 vs. right STN contacts 2–1, and left STN contacts 1–0 vs. right STN contacts 1–0. To minimize the false connectivity arising from volume conduction, the imaginary part of coherence was calculated as true functional coupling between bilateral STNs during VMC and VMR tasks (Nolte et al., 2004). Time-dependent imaginary coherence was calculated by integrating 1 s time windows across 30 trials (i.e., total time length was 30 s). Time-dependent coherence was then averaged across couplings and computed from −2 s to 4 s after the onset of VMC and VMR, in 0.05 s steps. The threshold for significant coherence at *p* < 0.05 was calculated based on the standard method developed by Halliday et al. (1995).

## Results

**Figure 1:**
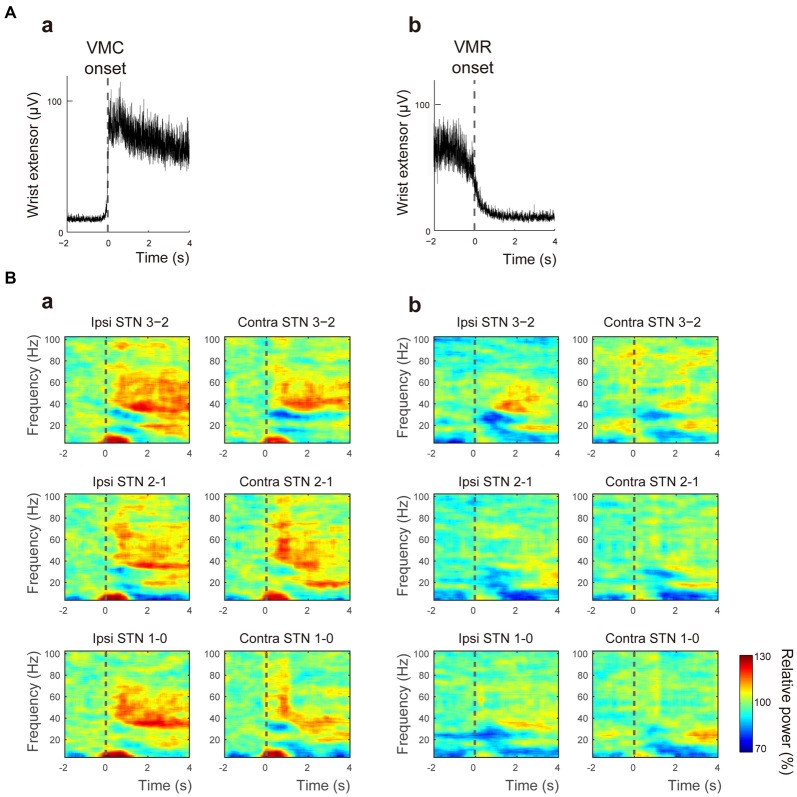
**Muscle activity and the time-frequency map of oscillatory activity in the bilateral subthalamic nucleus (STNs) related to voluntary muscle contraction (VMC) and voluntary muscle relaxation (VMR). (A)** The averaged activities of the wrist extensor muscle are shown, triggered at the onset of VMC **(a)** and VMR **(b)**. The dotted black bar represents the onset of VMC **(a)** and VMR **(b)**.** (B)** The time-frequency map of relative power in the bilateral STNs in each contact pair (i.e., Contacts 0–1, 1–2, and 2–3) is presented over the theta (4–7 Hz), alpha (8–13 Hz), beta (14–35 Hz), and gamma (40–100 Hz) bands in response to VMC **(a)** and VMR **(b)**. The dotted black bar represents the onset of VMC **(a)** and VMR **(b)**. Overall, alpha/beta desynchronizations are evident in each contact of the bilateral STNs in both VMR and VMC tasks. Theta/gamma synchronization also appears, especially in the VMC task.

In this study, the movement-related STN oscillations and their functional couplings between bilateral STNs during externally paced VMC and VMR of the wrist were investigated in seven PD patients. During the movement task, no involuntary movements such as tremors were observed over the recorded muscles in any patient. The activities of the wrist extensor were modulated in response to the onset of VMC (Figure 1Aa) and VMR (Figure 1Ab). The response times for the onset of VMC and VMR were 327.1 ± 83.1 ms and 363.8 ± 121.5 ms in the VMC and VMR tasks, respectively. Following VMC and VMR, the power in the bilateral STNs recorded from each adjacent contact pair (i.e., Contacts 1–0, 2–1, 3–2) showed frequency-specific ERS or ERD over the theta (4–7 Hz), alpha (8–13 Hz), beta (14–35 Hz), and gamma (40–100 Hz) bands across bilateral STNs around the time of onset of VMC (Figure 1Ba) and VMR (Figure 1Bb). Overall, alpha and beta ERDs were clearly observed in each contact of the bilateral STNs in both the VMR and VMC tasks. At the same time, theta and gamma ERSs also appeared especially in the VMC task. We observed no significant changes in the magnitudes of ERD or ERS among the contact pairs of Contacts 3–2, 2–1, and 1–0 (0–1 s after the onset of VMC or VMR). To further characterize the observed frequency-specific ERD and ERS, the time-dependent power changes in the bilateral STNs were compared between the phases of VMC and VMR, divided into the alpha and beta ERDs and the theta and gamma ERSs, as below.

### ERD in the Alpha and Beta Frequency Bands

**Figure 2:**
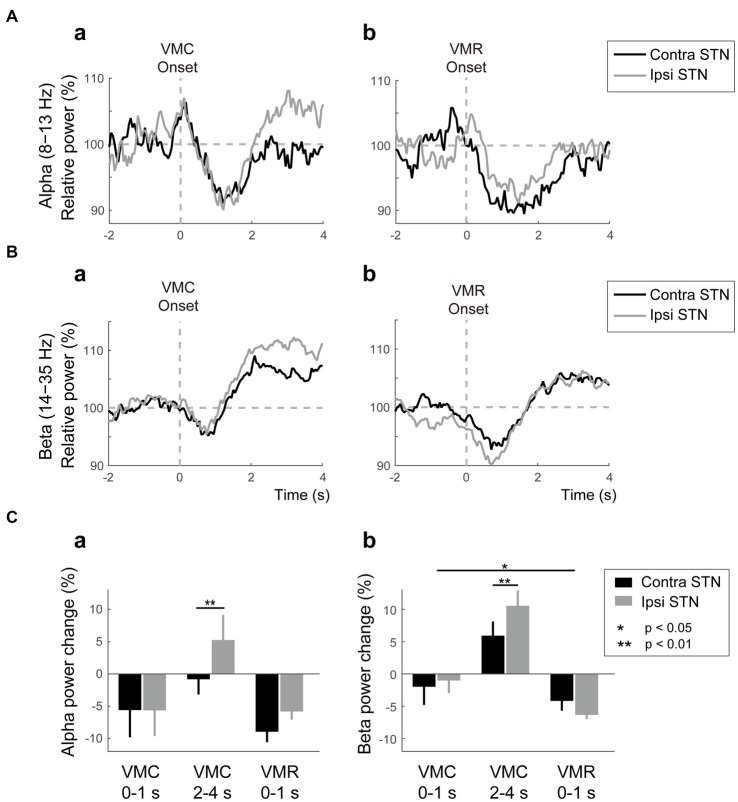
**Alpha/beta VMC- and VMR-related power changes in the bilateral STN. (A)** The averaged power in the contralateral STN (black) and ipsilateral STN (gray) over the alpha band is plotted before and after the onset of VMC **(a)** and VMR **(b)**. The vertical gray dotted line represents the onset of VMC **(a)** and VMR **(b)**. The horizontal gray dotted line represents 100% of the relative power in the bilateral STNs. Overall, the alpha event-related desynchronization (ERD) consistently appears in bilateral STNs in response to VMC and VMR. **(B)** The averaged power in the contralateral STN (black) and ipsilateral STN (gray) over the beta band is plotted before and after the onset of VMC **(a)** and VMR **(b)**. The vertical gray dotted line represents the onset of VMC **(a)** and VMR **(b)**. The horizontal gray dotted line represents 100% of the relative power in the bilateral STNs. Overall, the beta ERD and subsequent event-related synchronization (ERS) appear in bilateral STNs in response to VMC and VMR. **(C)** The magnitudes of the synchronization and desynchronization in the contralateral STN (black) and the ipsilateral STN (gray) in each of the selected time periods (i.e., 0–1 s before VMC, 2–4 s after VMC, and 0–1 s after VMR) are shown in the alpha **(a)** and beta **(b)** bands. The asterisks (*,**) indicate a significant difference in relative power. Error bars represent standard errors.

The averaged powers in the bilateral STNs across patients and contacts (i.e., Contacts 0–1, 1–2, and 2–3) in the alpha and beta bands were plotted against the onset of VMC and VMR (Figures 2A,B). The alpha ERD was observed bilaterally in both VMC and VMR tasks (Figures 2Aa,b,Ca). The magnitude of the alpha ERD was not different between ipsilateral and contralateral STNs in the VMC and VMR tasks. In the ipsilateral STN, however, the alpha ERD after VMC was shifted to ERS after 2 s of VMC (*p* < 0.05, Figure 2Ca).

In the beta band, ERD and the subsequent ERS (often described as “beta rebound”) were bilaterally observed after the VMC and VMR tasks (Figures 2Ba,b). The magnitudes of ERDs in the bilateral STNs 1 s after onset were greater in the VMR than in the VMC task (*p* < 0.05, Figure 2Cb). On the other hand, the beta rebound 2–4 s after VMC onset was greater in the ipsilateral STN than in the contralateral STN (*p* < 0.05, Figure 2Cb). Overall, the alpha and beta ERDs in bilateral STNs appeared consistently after the VMC and VMR tasks.

### ERS in the Theta and Gamma Frequency Bands

**Figure 3:**
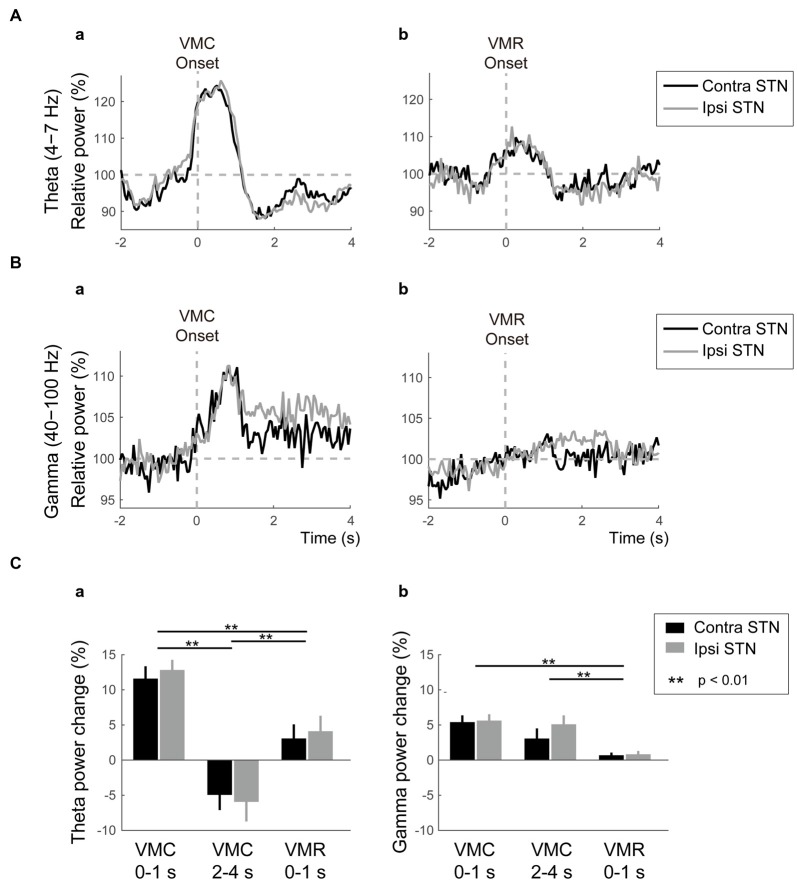
**Theta/gamma VMC- and VMR-related power changes in the bilateral STNs. (A)** The averaged power in the contralateral STN (black) and ipsilateral STN (gray) over the theta band is plotted before and after the onset of VMC **(a)** and VMR (b). The vertical gray dotted line represents the onset of VMC **(a)** and VMR **(b)**. The horizontal gray dotted line represents 100% of the relative power in the bilateral STNs. Overall, the theta ERS in bilateral STNs is more prominent in response to VMC than VMR. **(B)** The averaged power in the contralateral STN (black) and ipsilateral STN (gray) over the gamma band is plotted before and after the onset of VMC **(a)** and VMR (b). The vertical gray dotted line represents the onset of VMC **(a)** and VMR **(b)**. The horizontal gray dotted line represents 100% of the relative power in the bilateral STNs. Overall, the gamma ERS in bilateral STNs is evident only in the VMC task. **(C)** The magnitudes of the synchronization and desynchronization in the contralateral STN (black) and the ipsilateral STN (gray) in each of the selected time periods (i.e., 0–1 s before VMC, 2–4 s after VMC, and 0–1 s after VMR) are shown in the theta **(a)** and gamma **(b)** bands. The asterisks (**) indicate a significant difference in relative power. Error bars represent standard errors.

As well as the ERD in the alpha and beta bands, ERS in the theta and gamma bands emerged in the bilateral STNs after the VMC and VMR tasks, as shown in Figure 3. In the theta band, the ERS in bilateral STNs occurred transiently just around the onset of the VMC and VMR tasks (Figures 3Aa,b). However, the magnitude of theta ERS was greater in the VMC than in the VMR task (*p* < 0.01, Figure 3Ca), although these were not significantly different between the ipsilateral and contralateral STNs in each task (Figure 3Ca).

In the gamma band, the ERS was clearly observed in the bilateral STNs after the VMC task, but not after the VMR task (Figures 3Ba,b,Cb). Unlike the transient increase in the theta ERS, the gamma ERS after VMC increased continuously over 4 s after the onset. The magnitude of gamma ERS in the VMC task was not significantly different between the ipsilateral and contralateral STNs (Figure 3Cb).

Overall, theta ERSs were bilaterally present in the VMC and VMR tasks, but they were more prominent in the VMC than in the VMR tasks. On the other hand, the bilateral gamma ERSs were continuously observed only in the VMC task.

### Theta-Gamma and Beta-Gamma Phase-Amplitude Coupling

The time-dependent power changes in bilateral STNs clearly showed that the theta/gamma ERSs occurred simultaneously in response to the onset of the VMC and VMR tasks. In such cases, the ERSs in the theta and gamma bands may interact with each other via cross-frequency coupling, as previously demonstrated in the corticostriatal axis during motor behavior in rats (von Nicolai et al., 2014). To investigate the movement-related PAC over broad frequency bands, the PAC between the phases of low-frequency bands (i.e., theta, alpha, beta bands) and the amplitude of high-frequency bands (i.e., gamma band) were computed in bilateral STNs during the VMC and VMR tasks.

**Figure 4:**
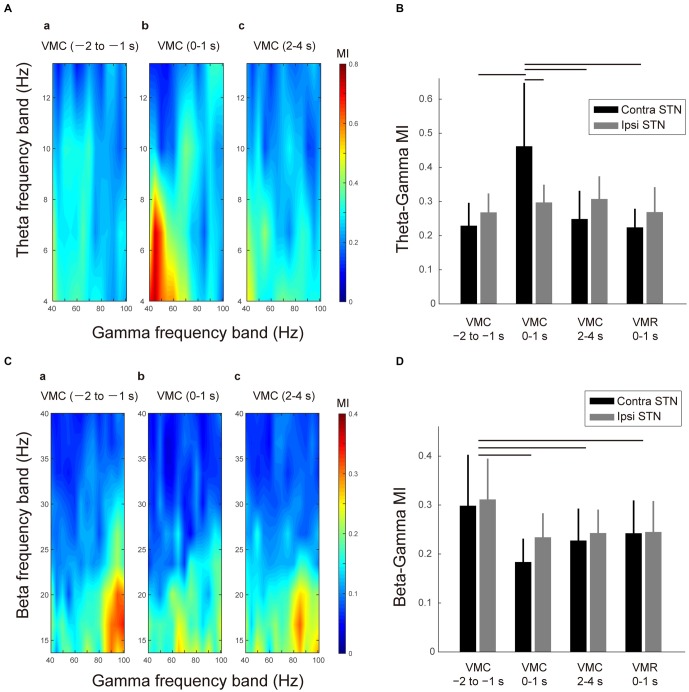
**Theta-gamma and beta-gamma phase-amplitude cross-frequency couplings (PAC) in the bilateral STNs related to VMC and VMR tasks. (A)** Representative color maps of the theta-gamma phase-modulation-index (MI) in the contralateral STN in the selected time periods before and after the VMC task (i.e., 1–2 s before VMC **(a)**, 0–1 s after VMC **(b)**, and 2–4 s after VMC **(c)**) are shown. The increase in the theta-gamma PAC can be observed after 0–1 s of the VMC onset. **(B)** The averaged MIs between the theta and gamma bands in the contralateral STN (black) and ipsilateral STN (gray) are plotted in the selected time periods of the VMC and VMR tasks (i.e., 1–2 s before VMC, 0–1 s after VMC, 2–4 s after VMC, and 0–1 s after VMR). Error bars represent standard errors. Horizontal bars indicate a significant difference in MI (*p* < 0.05). **(C)** The representative color maps of the beta-gamma MI in the contralateral STN in the selected time periods before and after the VMC task (i.e., 1–2 s before VMC **(a)**, 0–1 s after VMC **(b)**, and 2–4 s after VMC **(c)**) are shown. The decrease in the beta-gamma PAC after 0–1 s of VMC onset can be observed. **(D)** The averaged MIs between the beta and gamma bands in the contralateral STN (black) and ipsilateral STN (gray) are plotted in the selected time periods before and after the VMC task (i.e., 1–2 s before VMC, 0–1 s after VMC, 2–4 s after VMC, and 0–1 s after VMR). Error bars represent standard errors. Horizontal bars indicate significant difference in MI (*p* < 0.05).

A significant increase in theta-gamma PAC was found in the contralateral STN during 0–1 s after the VMC phase (Figure 4A). In particular, during this period of VMC phase, the theta phase was interacted with the amplitude of lower part of the gamma band (i.e., 40–60 Hz). Across patients and contact pairs (i.e., Contacts 0–1, 1–2, 2–3), the averaged phase-MI of theta-gamma PAC increased significantly in the contralateral STN during 0–1 s after VMC (Figure 4B). For the ipsilateral STN, we observed no significant change in theta-gamma PAC during VMC. On the other hand, in the VMR phase, significant changes in the theta-gamma PAC were not found in the bilateral STNs (Figure 4B), suggesting that the theta and gamma oscillations were jointly coupled via phase-amplitude cross-frequency interactions within the contralateral STN just around the onset of VMC.

In addition to the VMC-related change in the theta-gamma PAC, the beta-gamma PAC was also observed in the STN at rest (Figure 4Ca). In particular, the phase of the low beta band (i.e., 14–20 Hz) was interacted with the amplitude of higher part of the gamma band (i.e., 80–100 Hz) at rest. In addition, this beta-gamma PAC decreased in each time period of the movement phase of VMC (i.e., 0–1 s after VMC), the holding phase of VMC (i.e., 2–4 s after VMC), and movement phase of VMR (i.e., 0–1 s after VMR; Figures 4Cb,c). The decreases in the beta-gamma PAC during VMC and VMR tasks occurred bilaterally (Figure 4D).

Overall, the gamma oscillations were entrained by the beta phase at rest. However, this beta-gamma PAC decreased with VMC and VMR tasks. In particular, the gamma oscillations in the contralateral STN switched from being entrained from the beta phase to being entrained from the theta phase just after the VMC phase.

### Coherence Between Bilateral STNs

Similar features in the movement-related power changes in the bilateral STNs further suggested the synchronous relationship between ipsilateral and contralateral STNs over the selected frequency bands. These synchronizations may occur by functional coupling between bilateral STNs. To demonstrate the functional connectivity between bilateral STNs, the time-dependent change in coherence between ipsilateral and contralateral STNs in response to VMC and VMR onset was computed. To further characterize the directionality of the functional coupling between bilateral STNs, the time-dependent changes in Granger causality were evaluated.

**Figure 5:**
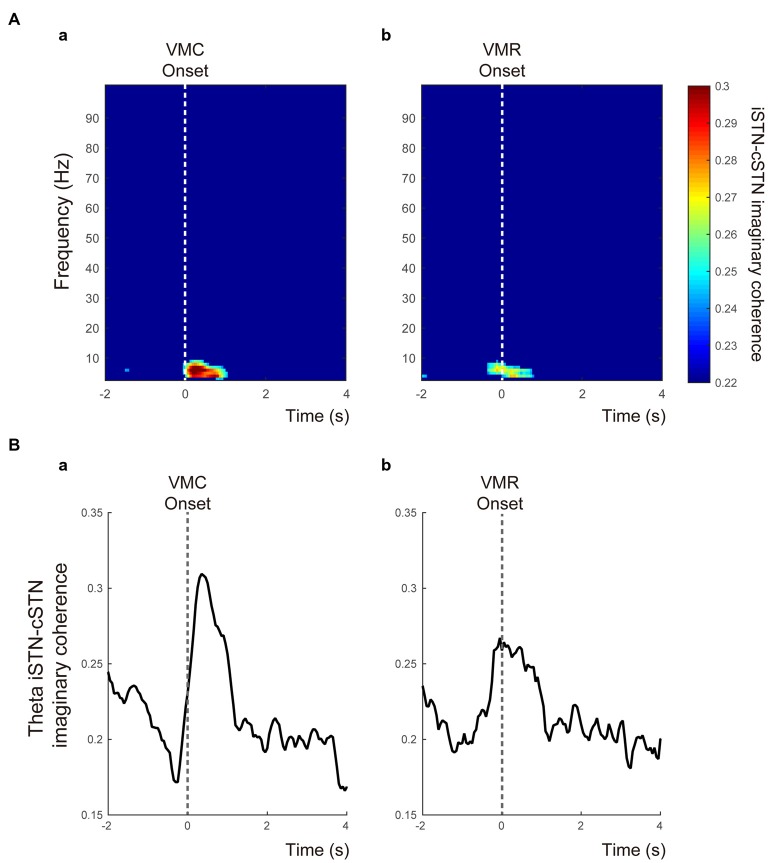
**Functional coupling between bilateral STNs in relation to the VMC and VMR tasks. (A)** The time-frequency maps of the time-dependent imaginary coherence between ipsilateral and contralateral STNs (i.e., iSTN-cSTN coherence) in the VMC **(a)** and VMR **(b)** tasks. Only significant coherence was visualized. The vertical white dotted lines show the onset of VMC **(a)** and VMR **(b)**. The theta coherence increases especially around the onset of the VMC task. **(B)** The averaged theta iSTN-cSTN coherence is plotted from 2 s before to 4 s after the VMC **(a)** and VMR **(b)** onset. The vertical gray dotted lines represent the onset of VMC **(a)** and VMR **(b)** tasks.

The time-frequency mappings of VMC- and VMR-related imaginary coherence between bilateral STNs were plotted (Figures 5Aa,b). These showed that the coherence between ipsilateral and contralateral STNs (iSTN-cSTN coherence) in the theta band increased transiently just around the onset of the VMC and VMR tasks (Figures 5Aa,b). In the other frequency bands, we found no significant coherence over time. The time-dependent change in theta coherence is shown in Figures 5Ba,b, suggesting that peaks of theta iSTN-cSTN coherence were more prominent after the onset of VMC than VMR (*p* < 0.05).

## Discussion

This study demonstrated that bilateral STNs modulated their frequency-specific oscillatory activities in response to VMC and VMR in PD patients. The alpha/beta ERDs in the bilateral STNs occurred during both VMC and VMR tasks, whereas the theta/gamma ERSs in the bilateral STNs were prominent specifically in the VMC task. Particularly, just after the VMC task, the theta functional coupling between the bilateral STNs increased transiently, during which the gamma activities in the contralateral STN were entrained by the theta phase via PAC. These results suggest that bilateral STNs are closely involved in the execution of both VMC and VMR through the theta-gamma networks between bilateral STNs.

### ERDs in Alpha and Beta Bands Over Bilateral STNs During Voluntary Muscle Contraction and Relaxation Tasks

In this study, the alpha/beta ERDs and subsequent ERSs occurred similarly in response to externally paced VMC and VMR tasks. Similar movement-related STN oscillatory changes in the alpha/beta bands have been extensively described in previous studies using VMC (Kühn et al., 2004; Loukas and Brown, 2004; Kempf et al., 2007; Tan et al., 2013) and VMR (Hsu et al., 2012; Tan et al., 2013) tasks in patients with PD. As with the self-paced movements, alpha/beta ERDs are generally believed to occur prior to the onset of the VMC (Cassim et al., 2000) and VMR (Hsu et al., 2012), suggesting that the STN has a feed-forward role in the voluntary control of muscle contraction and relaxation in patients with PD. In particular, the alpha/beta ERDs prior to the onset of VMR suggest that the STN plays an active role in the inhibition of on-going muscle contraction. We also confirmed that the antagonist wrist flexor was almost silent throughout the experiment. Thus, VMR-related oscillatory changes likely come mainly from relaxation of the wrist extensor and minimally from contraction of the wrist flexor muscle via reciprocal inhibition during VMR.

These movement-related alpha/beta ERDs also occur at least in the contralateral primary motor cortex and supplementary motor area before and during VMC (Toro et al., 1994; Ohara et al., 2000) and VMR (Labyt et al., 2006) tasks in healthy subjects. From these findings, the emergence of alpha/beta ERDs in the STN may reflect a physiological phenomenon, rather than being a characteristic marker in patients with PD. However, of note, in PD patients in the “OFF” medication state, the ERD in the alpha band starts later (Defebvre et al., 1996; Magnani et al., 1998), but becomes nearly normal in the “ON” medication state (Defebvre et al., 1998; Magnani et al., 2002) or when treated with STN DBS (Devos et al., 2004). In agreement with these studies, the present results during the “OFF” medication state showed a delayed response of alpha ERD in both the VMC and VMR tasks, supporting the idea that the delayed alpha ERD may reflect a pathophysiological feature of PD.

Additionally, alpha/beta oscillatory changes were present bilaterally in response to VMC as well as VMR, suggesting that the STNs were bilaterally involved with different unilateral motor programs of VMC and VMR. Indeed, as with VMR, previous studies have confirmed that in elderly healthy subjects compared to young healthy subjects, the distribution of alpha/beta ERDs is more widespread over the bilateral sensorimotor cortex, beginning earlier over contralateral frontocentral and parietocentral regions (Derambure et al., 1993; Labyt et al., 2004). These observations indicate that aging can also be associated with bilateralization of the alpha/beta ERDs, probably due to a compensatory strategy to correct the motor program. Moreover, another study demonstrated that even in healthy subjects, the alpha ERD recorded over the sensorimotor cortex by EEG or magnetoencephalograms (MEG) has contralateral preponderance during the early pre-movement phase, and then becomes symmetrically bilateral near the beginning of the movement (Derambure et al., 1993). These results may indicate that the motor commands for unilateral movement can be sent in a parallel processing manner between bilateral cortical levels. Anatomical evidence has demonstrated that the STN has rich connections to the motor cortex via cortical inputs to the striatum and the external segment of the globus pallidus via the “indirect pathway”, and without relay via the “hyper-direct pathway” (Alexander et al., 1986; Alexander and Crutcher, 1990; Nambu et al., 1996), whereas subthalamic output reaches the motor cortex via the ventrolateral thalamus (Hoover and Strick, 1999). As a consequence of these findings, a unilateral motor program such as VMC and VMR may be processed bilaterally both at cortical and subcortical levels such as the cortico-basal ganglia-thalamo-cortical loops across bilateral hemispheres.

### Synchronization in the Theta and Gamma Bands Over the Bilateral STNs

In addition to the alpha/beta ERD, the theta/gamma ERS was simultaneously observed specifically in response to the VMC task. The gamma ERS has been thought to arise from increasing asynchronous spiking activity of the STN local neuronal population (Ray et al., 2008; Manning et al., 2009; Miller et al., 2010; Ray and Maunsell, 2011), although another study demonstrated that the firing of neurons in the upper STN and the bordering zona incerta tends to be locked to gamma activity in the LFP (Trottenberg et al., 2006). In either case, the present results of VMC-specific theta/gamma ERS suggest that VMC may require more spike excitation than VMR, indicating that novel processes relevant to VMC and VMR were elicited in the bilateral STNs.

VMC-related theta/gamma ERS was also present bilaterally, as confirmed in previous studies (Alegre et al., 2005). However, the gamma ERS over the motor cortex, recorded from electrocorticography, was observed during movements in the contralateral side, but not the ipsilateral side (Crone et al., 1998). This result implies that VMC-specific theta/gamma oscillatory changes in bilateral STNs could be explained by bilateral cortico-striatal projections (Rouzaire-Dubois and Scarnati, 1985; Mouroux et al., 1995; Parent and Hazrati, 1995), assuming that the high gamma activity was generated primarily in the contralateral motor cortex in response to VMC.

One of the novel findings of the present study was that theta and gamma oscillations interacted with each other via cross-frequency coupling in the contralateral STN during the VMC task. This means that the phase of theta oscillation in the contralateral STN entrained the gamma amplitude, especially during the initiation of VMC. Originally, the idea of PAC between theta low frequency and gamma high frequency was thought to be a general mechanism for regulation of the neural ensemble in various functions such as working memory, visual perception, and reinforcement learning (Lakatos et al., 2005; Canolty et al., 2006; Siegel et al., 2009; Fujisawa and Buzsáki, 2011; Spaak et al., 2012; de Hemptinne et al., 2013). Recently, von Nicolai et al. (2014) have further shown that corticostriatal theta and gamma oscillations are strongly modulated via cross-frequency coupling by simple motor behavior in the rat, suggesting that the coordination of fast gamma oscillations through coherent phase-amplitude coupling may be a general mechanism for regulation of motor behavior. In excellent agreement with these studies, the present study first demonstrated that such theta-gamma cross-frequency coupling was also prominent within the contralateral STN and was especially related to the initiation of VMC in patients with PD.

In addition to movement-related theta-gamma PAC, we also confirmed movement-related decreases in the beta-gamma PAC in the bilateral STNs, as reported in previous studies (de Hemptinne et al., 2013, 2015). From the abundant evidence, the abnormal excessive beta oscillations in the STN at rest may be characteristic of the “OFF” medication state in PD (Brown, 2003; Bronte-Stewart et al., 2009; Kato et al., 2015). Thus, several previous studies have suggested that prominent beta-gamma PAC at rest reflects the inflexible and locked neural states in the STN, which may be related to parkinsonian motor impairments (de Hemptinne et al., 2013, 2015). After movement initiation, however, the neural constrained states due to the beta-gamma PAC over the bilateral STNs were temporarily canceled, and instead, the theta-gamma PAC emerged within the contralateral STN to accomplish VMC. To our knowledge, this is the first study to show that beta-gamma and theta-gamma PACs exist simultaneously in the STN in PD and are differentially modulated by VMC and VMR movements. However, whether the observed movement-related theta-gamma PAC in the STN plays a physiological role in regulation of voluntary movement remains unclear, because we recorded subthalamic activities in the “OFF” medication state. Therefore, the present findings may be specific to PD. Nevertheless, well-known anatomical evidence suggests that the striatum has rich connections to the STN via the “indirect pathway” and the “direct pathway”, as described above. Based on this evidence, the present findings may suggest that such a VMC-related increase in theta-gamma PAC may occur within the corticostriatal axis (von Nicolai et al., 2014), as well as the STN, to regulate motor behavior in a physiological manner, rather than being a pathophysiological biomarker in PD.

Additionally, theta functional coupling between bilateral STNs was found to increase just around the onset of VMC. As yet, no physiological evidence exists that shows a direct connection between bilateral STNs. From this evidence, bilateral functional coupling in the theta band may occur from the influence of a third nucleus other than the two STNs. For example, the thalamus is a candidate because it is well connected to multiple nuclei in the basal ganglia, and therefore, is capable of driving phase synchrony between bilateral STNs. Interestingly, although synchronized theta oscillation between the bilateral STNs was observed, theta-gamma cross-frequency coupling only emerged within the contralateral STN. The mechanism of the selected cross-frequency coupling between ipsilateral and contralateral STNs is still unclear. Further studies to elucidate this mechanism are needed. The present study, however, demonstrated that even though theta-gamma ERS occurred similarly in the ipsilateral and contralateral STNs, the theta-gamma interaction became selective to the contralateral STN just around the onset of VMC and may play a key role in the initiation of the movement.

### Study Limitations

Our experimental approach has some important limitations. First, the present findings are difficult to directly compare with normal subjects due to the invasive nature of LFP recordings in the STN. Recording in the “ON” medication state would have improved the generalizability. However, our research opportunity was limited during the “OFF” medication state in which the subthalamic activity would be severely impaired. Therefore, the present findings could be characteristic of PD. Although our study suggested that frequency-specific STN oscillatory changes are differentially involved in VMC and VMR in the “OFF” medication state in PD, further comparisons between the “ON” and “OFF” medication states will be necessary to explore the functional meaning of each frequency oscillatory change.

Second, although group level analysis was performed in this study because consistent results were found, the number of patients should be increased to allow more robust statistics, especially for the PAC and coherence analysis. As for the movement-related changes in PAC and coherence, these changes were demonstrated after we carefully checked for similar robustness in previous findings about VMC-related alpha/beta ERDs and theta/gamma ERSs. Therefore, we feel that our findings about PAC and coherence represent a generalizable phenomenon.

Lastly, from the anatomical perspective, the STN receives many types of inputs: GABAergic and inhibitory inputs from the globus pallidus, excitatory and glutamatergic inputs from the cerebral cortex in the motor cortex, and neuromodulatory inputs from dopaminergic axons from the substantia nigra pars compacta (Alexander et al., 1986 and Alexander and Crutcher, 1990). Considering these findings, the present results of the STN oscillatory patterns modulated by VMC and VMR may reflect the fact that individual types of excitatory and inhibitory synaptic connections contribute differentially to each of the executions of VMC and VMR. Of course, elucidating the functional meaning of each frequency-specific oscillation in this study is difficult, but will be clarified by combining the many different types of movement experiments, such as voluntary vs. passive movements, self-paced vs. externally paced voluntary movements, or movement with preparation vs. without preparation.

## Author Contributions

KK and FY designed research, KK and FY performed the experiment. FY, HI, TK, KH, AI, KK, RO, and MT performed the operation and treated patients pre-and post-operation. KK analyzed the data, KK, FY, JU wrote the article.

## Conflict of Interest Statement

The authors declare that the research was conducted in the absence of any commercial or financial relationships that could be construed as a potential conflict of interest.
